# Brainstem death due to intracranial hypertension with hydrocephalus, produced by a third ventricular colloid cyst

**DOI:** 10.1259/bjrcr.20220007

**Published:** 2022-03-16

**Authors:** Muhammad Shoyab

**Affiliations:** 1 Radiology & Imaging, Japan East West Medical College Hospital, Dhaka, Bangladesh

## Abstract

We describe the clinical presentation and CT findings of a 10-year-old female patient about half an hour prior to cardiorespiratory arrest and subsequent death, resulting from brainstem compression and herniation caused by hydrocephalus and intracranial hypertension, produced by a previously undetected colloid cyst occluding foramina of Monro on both sides. While third ventricular colloid cysts are rare lesions, sudden unpredicted deaths have been attributed to undetected colloid cysts in many case reports, with some authors even considering that 10% of colloid cyst patients suffer such ends. However, there is still no conclusive or comprehensive guideline regarding how to prevent such situations. We make a short literature review and put forward a few recommendations or learning points to that end.

## Case report

### Clinical presentation

A 10-year-old girl presented to the Emergency Room in semi-unconscious condition. She had been gradually losing consciousness and awareness since the previous night. Her parents also mentioned occasional complaints of headache and dizziness. CT was immediately advised, suspecting intracranial pathology as the cause of the sudden loss of consciousness.

### Imaging findings

**Figure 1.**
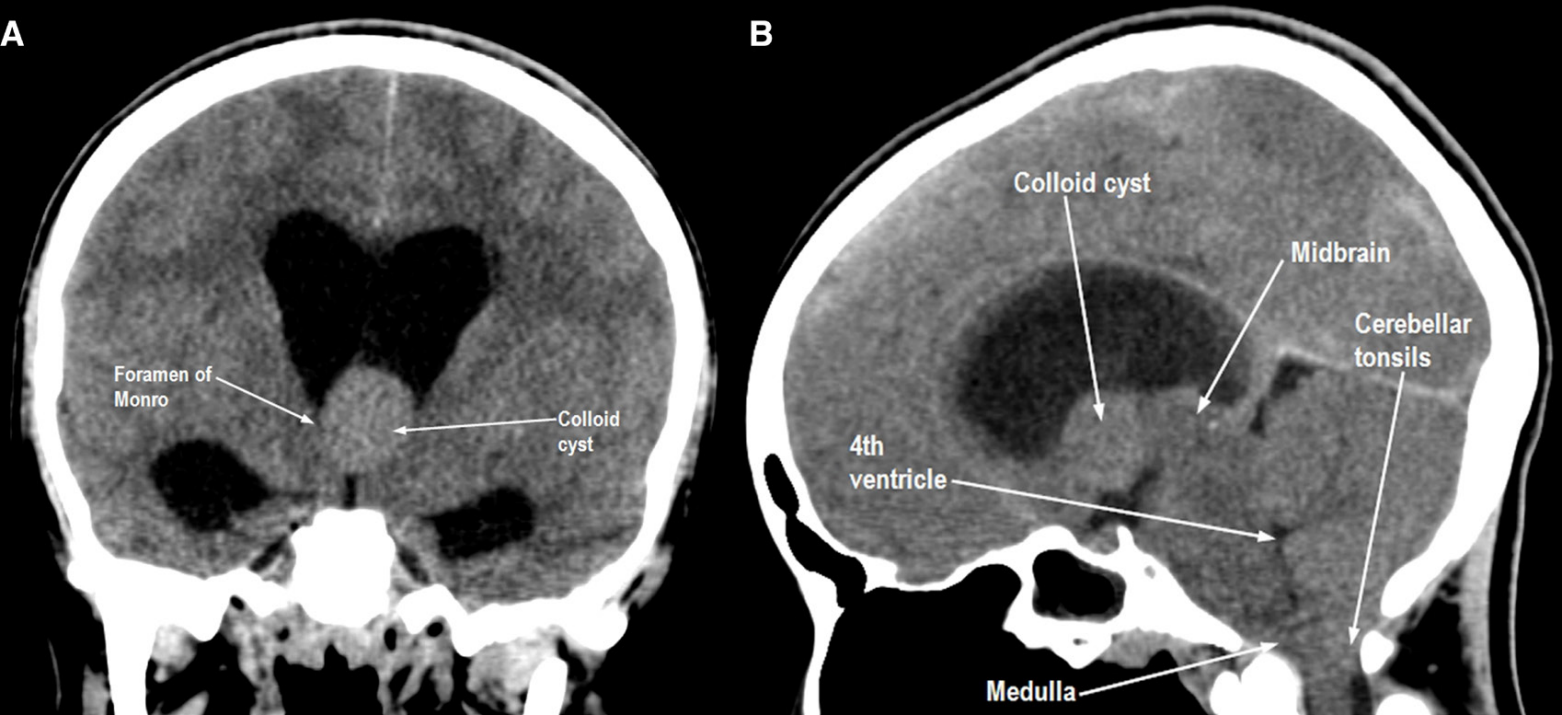
(a) 2 cm hyperdense mass (colloid cyst) occluding both foramina of Monro, producing dilatation of lateral ventricles bilaterally. Accentuation of gray–white interface with effacement of all subarachnoid spaces all over. (b) Cerebellar tonsils herniated below basiocciput, effacing pre-medullary cistern, producing medullary compression. Entire brainstem swollen and hypodense, effacing fourth ventricle, cerebral aqueduct and all basal cisterns. Brainstem herniated upwards above level of upper surface of cerebellum.

Plain CT scan revealed a hyperdense mass (2 × 1.5 x 1.5 cm) in the third ventricle, occluding both foramina of Monro (Figure 1a), producing dilatation of lateral ventricles and their horns bilaterally. A thin layer of periventricular hypodensity representing cerebrospinal fluid seepage was also seen. Accentuation of gray–white contrast with effacement of subarachnoid spaces all over both cerebral and cerebellar hemispheres (Figure 1a and b) indicated vasogenic edema. Both cerebellar tonsils were herniated 7 mm below the foramen magnum, producing effacement of the pre-medullary cistern along with medullary compression (Figure 1b). The entire brainstem was swollen with hypodense appearance, representing edema and effacing all basal cisterns, fourth ventricle and cerebral aqueduct (Figure 1b). Brainstem was also herniated upwards above tentorial level (Figure 1b), with the swollen midbrain compressed between thalami and temporal lobes from both sides. Both optic nerves were tortuous, with downward bowing of optic chiasma and optic tracts, representing intracranial hypertension.

### Treatment and outcome

The patient suffered cardiorespiratory arrest on the CT table, and was rushed back to the Emergency Room. There, she received CPR, thereafter immediately intubated and subsequently shifted to artificial life support. Intravenous steroids and mannitol were administered to reduce cerebral edema. After conclusive diagnosis of brainstem death based on clinical and CT features, her guardians were explained about her condition and prognosis. They consented to withdrawal of life support after 24 hours, and the patient expired shortly afterwards. Post-mortem was not done, since it was not a legal requirement in this case.

## Discussion

While it has been known for at least 50 years^1^ that third ventricular masses can produce sudden death in 10% cases,^2^ there is still no consensus^3^ regarding how to avoid such deaths. One reason for the inconclusiveness may be that colloid cysts are statistically rare and *per se* benign and slow growing. Another reason may be the hesitancy to advise CT or MRI for the vague symptoms of these patients, *e.g*. headache, dizziness, vertigo, blurred vision etc, since imaging is the only definitive tool for identifying a third ventricular lesion. Although it is commonly understood that finding papilloedema on fundoscopy may point towards intracranial hypertension produced by a mass lesion or hydrocephalus, there are studies^4^ which found that >90% of such subjects, *i.e*. individuals having headache, vertigo, dizziness etc exhibit no significant findings. This may be correlated with the pathogenesis of intracranial hypertension in third ventricular lesions, whereby the hypertension is only intermittent, due to the ball-valve nature of the occluding mass.^5^ The colloid cyst risk score (CCRS)^6^ may stratify high-risk patients who would require surgery, but this score is mainly based on imaging findings and intended for surgical screening. It comprises a total of five parameters (two clinical and three imaging features), and one point is scored in presence of each of them. The points are : age <65 years, headache, lesion diameter ≥7 mm on axial images, lesion location in “risk zone” for foraminal obstruction, hyperintense appearance of the lesion on FLAIR images. Scores 4 and 5 indicate high-risk and require surgery, while 3 indicates intermediate risk.^6^ Our patient has a CCRS of 4 (=high risk) on the CT, which could also be 5 out of 5 if she got an MRI, but that is only after the imaging is done. Without imaging, the two non-imaging features of the CCRS are positive in thousands of individuals, including our patient. Thus, it is not a good guide for deciding which patients should be evaluated further by fundoscopy or imaging.

Therefore, in order to prevent sudden, unpredicted deaths by an undetected third ventricle lesion while avoiding unnecessary fundoscopy or imaging, we recommend that the relevant clinical algorithms or scoring systems for headache, vertigo, blurred vision, seizures etc incorporate distinct indications regarding when to do fundoscopy and when to advise imaging.

Informed written consent was obtained from the patient’s guardian.

## Learning points

- Meticulously follow relevant guidelines or scoring systems before classifying a child/adolescent’s headache as migraine or idiopathic.
- Do fundoscopy and/or counsel for imaging when in slightest doubt.
- Aware guardians that their child’s intermittent headache, visual disturbance etc may have causes other than eye problems or migraine.
